# Immunohistological staining of unknown chemokine RANTES/CCL5 expression in jawbone marrow defects—osteoimmunology and disruption of bone remodeling in clinical case studies targeting on predictive preventive personalized medicine

**DOI:** 10.1007/s13167-019-00182-1

**Published:** 2019-08-06

**Authors:** Johann Lechner, Tilman Schulz, Volker von Baehr

**Affiliations:** 1 Clinic for Integrative Dentistry, Grünwalder Str. 10A, 81547 Munich, Germany; 2 Institute of Pathology, Klinikum Bayreuth, Preuschwitzer Sr. 101, 95410 Bayreuth, Germany; 3 Department of Immunology and Allergology, Institute for Medical Diagnostics in MVZ GbR, Nicolaistr. 22, 12247 Berlin, Germany

**Keywords:** Predictive preventive personalized medicine, Osteoimmunology, Osteonecrosis of the jawbone, Bead-based Luminex® analysis, Hyperactivated signaling pathways

## Abstract

**Background:**

Fatty degenerative osteonecrosis in the medullary spaces of the jawbone (FDOJ) may be identified as a lesser known source of RANTES/CCL5 (R/C) overexpression. The chemokine R/C also interferes with bone metabolism leading to osteolysis in areas affected by FDOJ. Many dental surgeries require functioning repair mechanisms and these may be disrupted by R/C overexpression.

**Objective:**

To clarify the way in which R/C expression from adipocytes in FDOJ causes a disturbance in osteogenesis and impacts on medullary stem cells by investigating the detection of R/C expression with immunochemical staining.

**Materials and methods:**

We examined the tissue samples of 449 patients with FDOJ to assess the level of the chemokine R/C using bead-based Luminex® analysis. In six clinical case studies of FDOJ, we compared bone density, histological findings, R/C expression, and immunohistochemical staining.

**Results:**

R/C is overexpressed by up to 30-fold in the 449 FDOJ cases when compared with healthy jawbone samples. The comparison of the six clinical cases consistently shows greatly reduced bone density, (i.e., osteolysis), but varies in terms of the level of agreement across the other three parameters.

**Discussion:**

R/C from FDOJ sources may be implicated in several immune responses and considered a key pathogenetic pathway for increased adipogenesis rather than desirable osteogenesis. Adipocytes pathogenetically act via R/C expression in local FDOJ and systemically on the immune system.

**Conclusion:**

R/C may be regarded as an important trigger for possible pathological developments in the fate of hematopoietic stem cells. FDOJ is not a rigidly uniform process but reflects changing stages of development. The absence of correlating findings should not be interpreted as a misdiagnosis. It seems appropriate to direct further research in the field of “maxillo–mandibular osteoimmunology” focusing on R/C overexpression in FDOJ areas. This may contribute to the development of personalized strategies in preventive medicine.

## Background

Bone marrow consists of different areas with varying cellular functions. On the one hand, stromal or mesenchymal cell systems (MSCs) are sources of osteoblasts (OBs), fibroblasts, and fat cells; on the other, the hematopoietic system fills the spaces within the marrow stroma and exerts a significant influence on the immune system. In the jawbone, the long-term success of any oral surgery depends on the interactions of these two different cell systems which significantly control the regeneration process and are essential components of bone metabolism. Osteoclasts (OCs) and OBs determine bone mass as well as the structure and strength of bone through their respective roles in bone resorption and formation. Bone remodeling is a spatially coordinated, lifelong process in which an old bone is removed by OCs and replaced with bone-forming OBs. The refilling of cavities which form due to resorption is incomplete in many pathological conditions, resulting in a net loss of bone mass during each remodeling cycle. A unique bone remodeling situation appears to occur in the case of fatty degenerative osteonecrosis in the medullary cavity of the jawbone (FDOJ), as previously described by our team and other authors [1, 2]. Since any manipulation of the jawbone—e.g., implantation, tooth, and wisdom tooth removal—activates multiple inflammatory processes, this is followed by physiological bone repair mechanisms.

### What is FDOJ? Morphological presentation of a fatty degenerative change in the jawbone

**Fig. 1:**
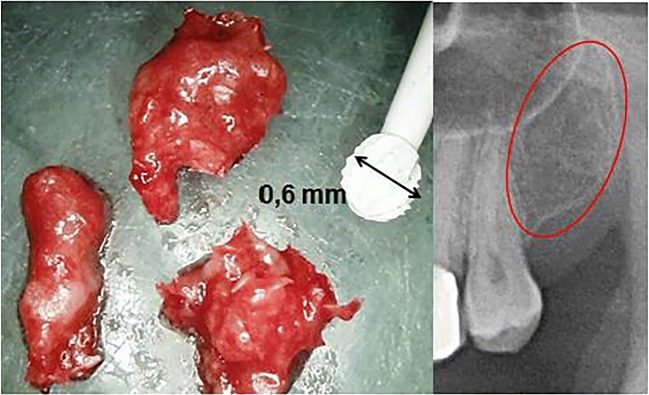
Left panel: Fatty and osteolytic spongy bone samples of medullary jawbone. Right panel: The corresponding radiograph of an apparently unaltered area of the upper left jawbone (28/29)

Chronic inflammation strongly influences osteoimmunology, thereby determining profound metabolic, structural, and functional changes in the bone [3]. Reports of so-called medullary edema of the jawbone and circumscribed “focal” bone defects in the jawbone (“focal osteoporotic marrow defects”) are frequently described and discussed in the scientific literature [4, 5]. Jawbone cavitations are characterized by dying or dead bone marrow fractions. These fatty degenerative osteolyses of the jawbone (FDOJ) can be painful or remain asymptomatic for years. Areas affected by FDOJ may be impacted by the overexpression of inflammatory mediators, specifically R/C [1, 6, 7]. The macroscopic features of FDOJ bone samples are consistently similar. Due to the softening of the bony substance, the marrow space can be curetted. Degeneration of cancellous bone extends into the mandibular areas, reaching as far back as the canal of the inferior alveolar nerve. Our team has documented the severity of these lesions in previous publications [1, 6]. Figure 1 shows an FDOJ specimen that exhibits a predominantly fatty transformation of the jawbone as well as the corresponding radiograph which shows an apparently unaltered area of the upper left jawbone.

### Specific RANTES/CCL5 overexpression in FDOJ and possible connection to systemic diseases

The inflammatory response of adipose tissue associated with a systemic inflammatory response is well known and widely discussed. Secretion of inflammatory cytokines mediates the systemic effects of adipose tissue inflammation [8]. Most normal adult tissues contain few, if any, R/C positive cells. In contrast, R/C expression dramatically increases in inflammatory sites [9]. These results indicate a wider expression of R/C than previously appreciated and suggest multiple physiologic roles for this mediator as found typically in FDOJ. Reduced blood flow and capillary density followed by ischemia in the medullary spaces of jawbone may lead to a hypoxic situation [10]. These triggers lead to the activation of signaling pathways which favor a predisposition to the development of chronic disease. In general, cell communication systems are organized as cascades with sequenced stages [11]. Signaling messengers like cytokines carry instructions and are received by those cells with specific receptors which are able to recognize them. In earlier publications, we defined this chronic inflammatory process as fatty degenerative osteonecrosis in the medullary spaces of the jawbone (FDOJ) [12, 13]. This clinical material was obtained from patients who were surgically treated at our clinic for suspected chronic inflammation in the jaw area. FDOJ was supposed to contribute to the systemic inflammatory diseases. Proinflammatory signaling mediators such as R/C in particular affect the organism systemically and may result in chronic inflammatory processes. It is generally accepted that an imbalance between cytokines and their specific inhibitors is characteristic of chronic inflammatory conditions [14]. Cytokines merge to initiate an immune response and induce acute inflammatory events in the case of persistent chronic inflammation (CI). This means that in order to maintain healthy conditions cytokine-producing mechanisms must be controlled [15]. FDOJ represents a new inflammatory cellular response phenomenon in that the cytokines are not triggered by the presence of bacteria or viruses. Accordingly, we have hypothesized that R/C signaling is a chronic disturbance typically deriving from FDOJ areas that may contribute to the development of CI.

## Objectives

The aim of this work is to explore the bone-resorbing processes of FDOJ and examine the local and immunological functions of any accompanying RANTES/CCL5 (R/C) overexpression. The question posed here is whether there is a common pattern in the mediator expression in FDOJ areas. We specifically explore our clinical findings in order to examine whether there are differences in R/C expression across different stages of FDOJ development, i.e., to determine whether FDOJ is a stable state, or a process of individually changing phenomena. Thus, the aim of this study is to demonstrate the radiographic, histopathological, immunohistochemical, and mediator-based conditions of the interactions between disturbed bone remodeling in FDOJ and the immune system according to a personalized and individually tailored treatment.

## Materials and methods

To target our objectives, we examined two different cohorts: the first group consisted of 449 patients with clearly defined bone marrow defects proved by low bone density measured by Hounsfield units. In all these areas, we found FDOJ. Each FDOJ sample was analyzed by histology, RANTES/CCL5 (R/C) expression and immunohistological staining. Unlike to our expectations, some samples showed uneven results in these postoperative findings. To enlighten these deviant findings, we selected six patients out of the first group for a detailed examination of their individual personalized patterns (see “Clinical cases (group 1) from the jaw area—comparison of X-ray diagnostics, R/C expression, light microscopy, and immunohistochemistry” section).

### Study participants

This study is based on data retrieved from patients during routine dental surgery; hence, no specific inclusion criteria were applied except for the fact that all patients suffered from chronic inflammation of the jawbone. The clinical case studies presented herein were performed as part of a case-control study and were deemed to be retrospective in nature. Approval was granted by IMD-Berlin forensic accredited institute DIN EN 15189/DIN EN 17025. All patients provided their written consent (as outlined in the PLOS consent form) to participate in the studies, and the samples and data were collected in the course of daily routine practice, i.e., regular oral surgical procedures during normal medical treatment. As shown in “What is FDOJ? Morphological presentation of a fatty-degenerative change in the jawbone” [1, 2] and “Specific RANTES/CCL5 overexpression in FDOJ and possible connection to systemic diseases” [3, 8–15]. R/C overexpression propels many diseases connected to CI. By the curettage of the FDOJ cavity, our dental treatment consists in minimizing the conspicuous R/C inflammatory signaling for the benefit of the patient.

### X-ray analysis of fatty degenerative changes in the jawbone—Hounsfield units and diagnosis of bone marrow defects

The existence of FDOJ has remained largely neglected in mainstream dentistry. The reason for this is that conventional X-ray techniques are limited in their ability to visualize areas affected by FDOJ [5]. Given these diagnostic difficulties, FDOJ is often underdiagnosed by dentists. Modern X-ray methods, which include digital volume tomography (DVT) and cone-beam computed tomography (CBCT), enable the clinician to perform a three-dimensional assessment of the jawbone. This also allows bone density to be assessed via computed tomography (CT) using CT values in Hounsfield units (HU). SimPlant software measurements of bone mineral density that were conducted in the posterior mandible (3D Diagnostix, Boston, MA, USA) illustrated that the mean CT value was 669.6 HU [16]. Further investigations classified the cancellous bone density of the jawbone into five categories, where the worst bone density for normal jawbone, (i.e., class 5), is < 150 HU. Thus, the HU values (range < 150 to − 370) produced in our study indicate osteolysis of the jawbone in class 5 cases (see Fig. 11 in “Summary of Results”) [17]. The device used by our team for DVT diagnostics was Orange 3D PaX-i3D duo Multi X-ray which showed the HU values over a selected area of jaw. The HU values of the clinical cases presented here are displayed in two-dimensional orthopantomogram (OPG) images.

### Histological presentation of fatty degenerative changes in the jawbone

Each FDOJ bone sample was examined under light microscopy with standardized hemalaun and eosin stain.

### Multiplex examination of 449 FDOJ samples for RANTES/CCL5 expression

To clarify the interactions between fat cells and the mediators they express, we have analyzed FDOJ samples obtained from the upper and lower jaws, (i.e., from the distal bone marrow sections), with R/C expression multiplex analysis in 449 patients since January 2017 (IMD-Berlin Nicolaistrasse 22 12247, Berlin, Germany). Bisphosphonate medication was the central exclusion criteria. This study cohort of 449 patients was comprised of five groups of patients with various specialist-diagnosed ISDs: atypical facial and trigeminal pain (*n* = 91); neurodegenerative disorders (multiple sclerosis and amyotrophic lateral sclerosis) (*n* = 43); tumors (breast cancer and prostate cancer) (*n* = 53); rheumatism (fibromyalgia and Lyme disease) (*n* = 166); and chronic fatigue syndrome (*n* = 96). The patients’ average age was 53.02 years (age range 22–74 years). There was a gender ratio (females to males) of 298:151.

As known from previous studies and publications [1], the chemokine R/C is almost exclusively present in FDOJ areas at up to 35-fold overexpression; as such, we decided to limit our investigation to this proinflammatory chemokine.

**Fig. 2:**
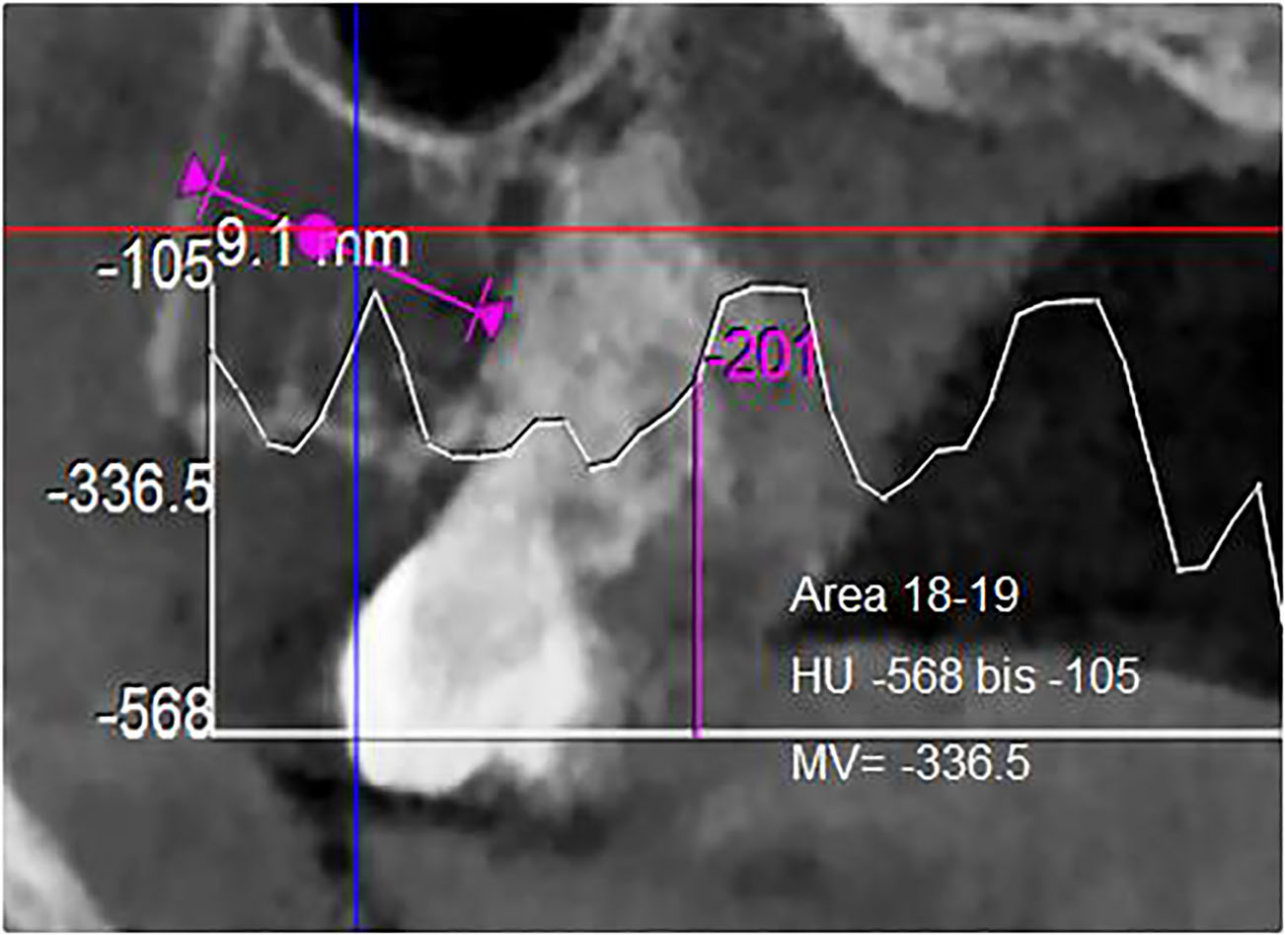
Example of a DVT-HU measurement in retromolar area 18 with evaluation; the HU attenuation coefficients are displayed over the entire test section as a progression curve (− 105 up to − 568). In this study, only the mean values (MV) are used (− 336.5)

In 468 samples of jawbone, (i.e., 449 FDOJ samples that were obtained from patients with systemic diseases or tumors and 19 healthy jawbone samples), R/C expression was measured. The demographic data of the 19 subjects in the healthy non-FDOJ control group were as follows: mean age, 51.4 years (range 33–72 years); sex, female/male ratio, 9/10. In the 449 FDOJ patients (age range 17–88 years; 68.5% female), the samples were typically obtained from the wisdom tooth area (area #8) and retromolar area (the so-called area #9). FDOJ lumps, (a clinical example of which is exhibited in Fig. 2), were scooped out up to a volume of 0.5 cm^3^ and these pea-sized lumps of tissue were immediately placed in a sterile collection vessel (Sarstedt Micro-Tube Ref. 72.692.005). The samples were hermetically sealed and stored at − 20 °C until transported to the laboratory. There, the tissue material was mechanically processed and taken up in 200 μL of protease buffer (Complete Mini Protease Inhibitor Cocktail, Roche, D) and homogenized. The homogenate was centrifuged for 15 min at 13,400 rpm; the supernatant was removed and centrifuged for an additional 25 min at 13,400 rpm. The determination of R/C was carried out in the supernatant of the tissue homogenate using the Human Cytokine/Chemokine Panel I (MPXHCYTO-60K, Millipore GmbH, Schwalbach, D) according to the manufacturer’s protocol and using Luminex 200™ with xPotent© software (Luminex, Austin, TX, USA).

### Immunohistochemistry of RANTES/CCL5 expression in FDOJ

Some FDOJ samples underwent immunohistochemistry analysis. For this analysis, 5-μm sections of poly-l-lysine-coated slides were used after the samples were dried in an oven, followed by dewaxing and peroxidase blocking with 1% H_2_O_2_. The specificity of immunostaining was tested by replacing primary antisera with normal rabbit serum. No additional pre-treatment procedures for antigen retrieval were conducted. The immunoreactions were performed with the following antibody and staining protocol: Anti-RANTES/CCL5 (polyclonal; Abcam; dilution 1:500; retrieval CC1 68′; incubation, Benchmark Ultra; chromogen, ultraView Red). The presence of clearly visible red precipitation was considered to be an immunoreaction product.

Immunohistochemistry studies show that most normal adult tissues contain few, if any, R/C-positive cells. In contrast, R/C expression increases dramatically in areas of inflammation [18]. Further, megakaryocytes, some tumors, and selected fetal tissues express high levels of R/C-positive cells. Here, we used selected polyclonal anti-RANTES antibodies for the immunohistological staining procedure of a number of FDOJ tissue samples (Schulz, T., Institut für Pathologie, Klinikum Bayreuth GmbH, Preuschwitzer Str. 101, 95445 Bayreuth, Germany). To our knowledge, this is the first time that R/C expression in the jawbone has been visualized and quantified using immunohistochemistry.

### Assessment of six clinical cases with bone marrow defects using four parameters

To perform individual comparisons of dental radiography, we employed light microscopy, multiplex analysis, and the immunohistochemistry of R/C expression in the areas affected by FDOJ, and compiled three groups of clinical cases from the entire collection of samples:

- Two clinical cases of FDOJ with similar histology and R/C overexpression;
- Two clinical cases of FDOJ with inconsistent histology, (i.e. normal findings), and R/C overexpression; and
- Two clinical cases of FDOJ with inconsistent findings in terms of both histology, (i.e., normal findings) and a lack of R/C expression.

### Statistical methods

Measurements of both FDOJ and control groups were subjected to descriptive statistical analyses. The medians, means, and distributions of the data were determined to assess whether non-parametric or parametric testing would be more appropriate for the analysis. Differences between cohorts were determined using Student’s *t* test or Spearman’s Rho. The significance level was set at *P* < 0.05.

## Results

### Multiplex analysis of 449 FDOJ cases: RANTES/CCL5 expression when compared with healthy jawbone

**Fig. 3:**
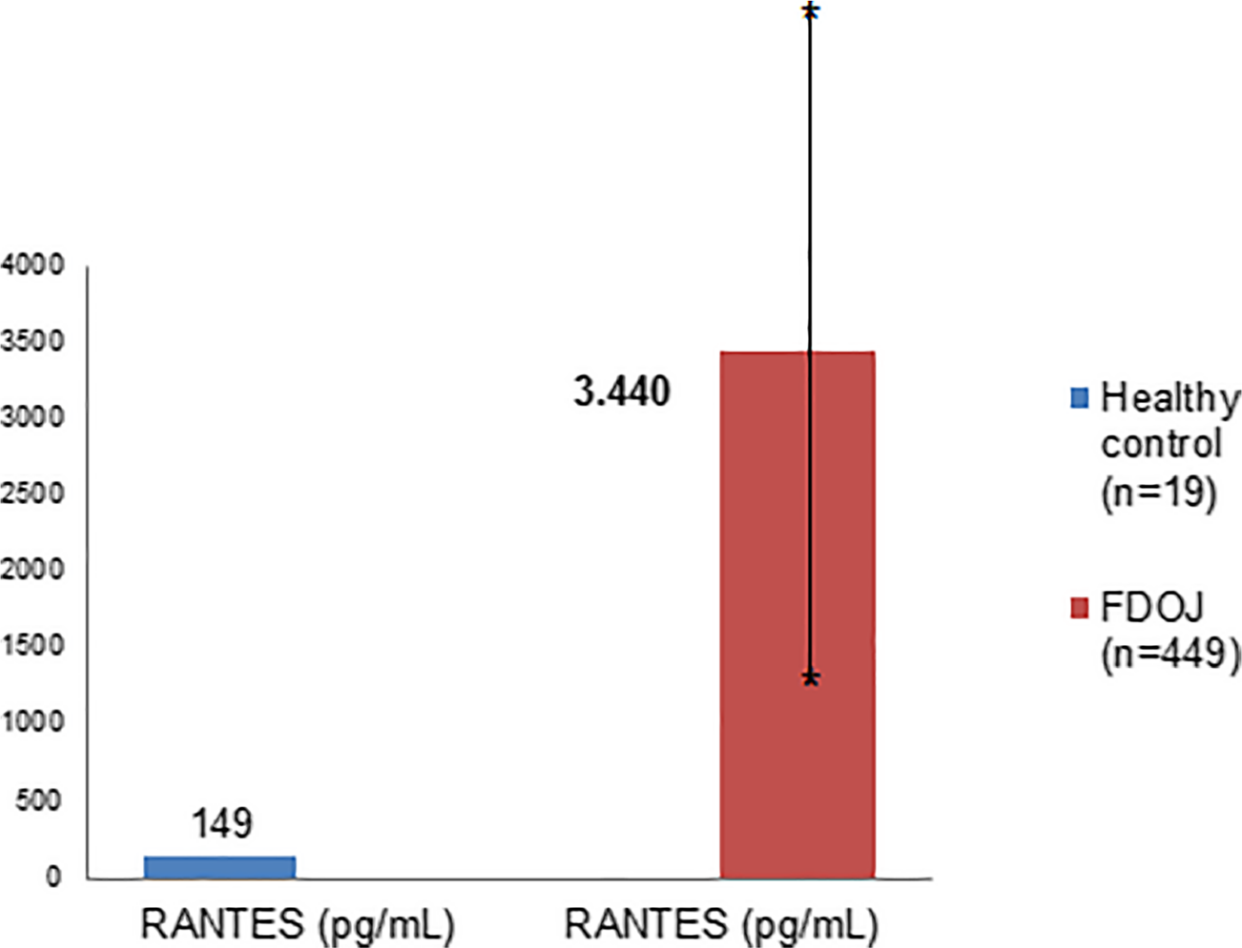
Distribution of RANTES/CCL5 expression in 449 FDOJ samples when compared with normal, healthy jawbone samples (*n* = 19)

The median R/C expression levels in the tissue samples of the FDOJ group (*n* = 449) and healthy jawbone (*n* = 19) are compared and summarized in Fig. 3. The FDOJ samples show a 22.9-fold expression of R/C at 3448 pg/mL (SD ± 4,487) as compared with the healthy control group at 149 pg/mL.

### Clinical cases (group 1) from the jaw area—comparison of X-ray diagnostics, R/C expression, light microscopy, and immunohistochemistry

All cases shown in “Clinical cases (group 1) from the jaw area—comparison of X-ray diagnostics, R/C expression, light microscopy, and immunohistochemistry –“Summary of results” share common features along with inconspicuous, two-dimensional X-ray diagnostics: they exhibit a positive diagnosis for FDOJ based on transalveolar ultrasonography (TAU)—a measurement of bone density, where bone density < 150 HU (class 5 HU) indicates highly significant osteolysis—and demonstrated noticeable softening and fatty degenerative changes in medullary morphology.

#### Case #1: Male, aged 55 years

##### Symptoms and clinical diagnosis: chronic fatigue syndrome

**Fig. 4:**
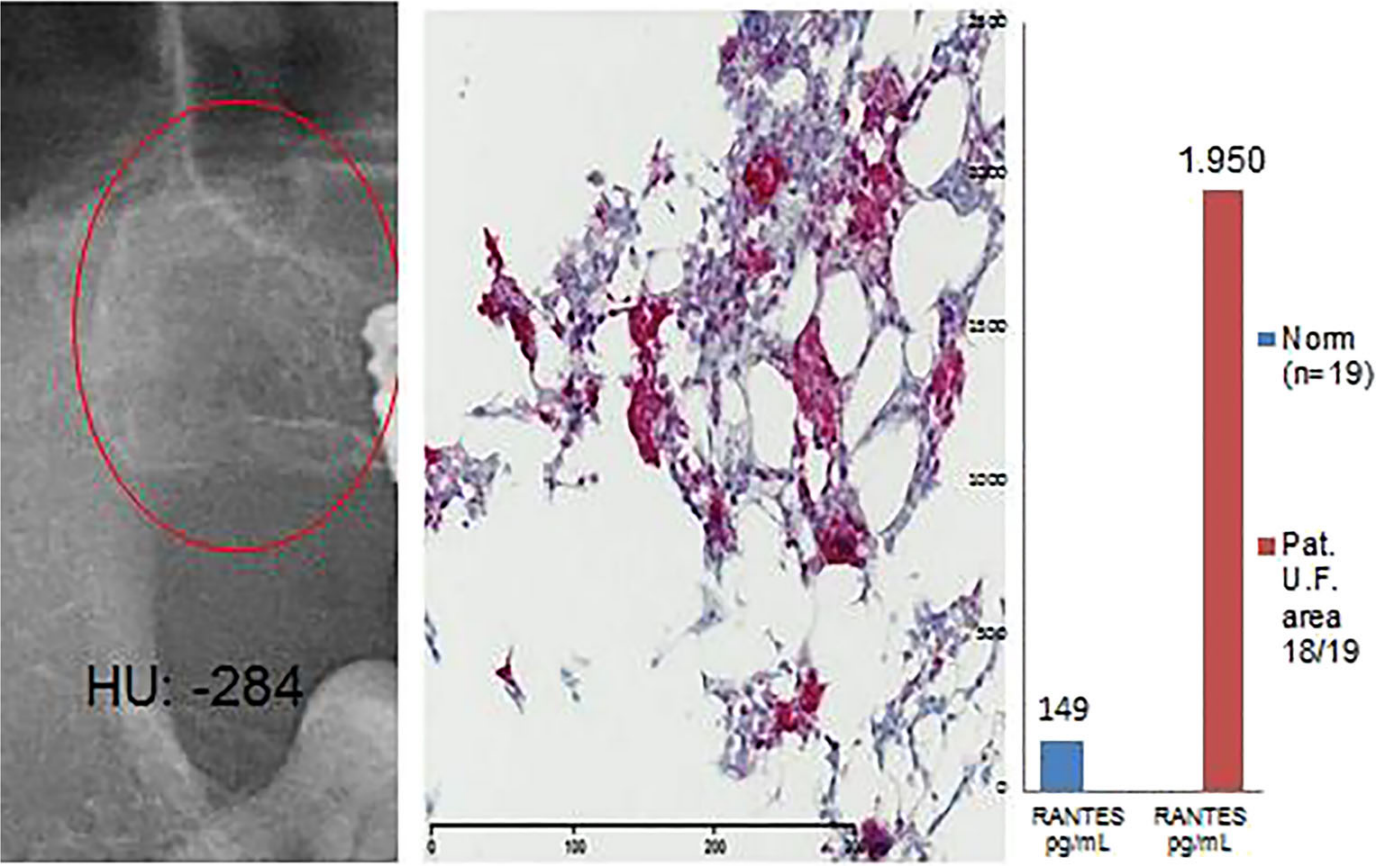
Left panel: Inconspicuous, two-dimensional X-ray of area 18/19 with a bone density of − 284HU. Middle panel: Immunohistochemistry shows a strong positive red staining for adipocytogenic R/C overexpression. Right panel: Multiplex R/C expression at a level of 1950 pg/mL (norm = 149 pg/mL)

Histological diagnosis and assessment: Fatty tissue from area 18/19 with occasional, loose marrow fibrosis and extended regions of fatty-degenerated marrow. The latter contains numerous, variably sized oil cysts with necrobiotic fat cell remnants, accompanied by a discrete, inflammatory round-cell reaction. These findings are suggestive of a diagnosis of FDOJ (Fig. 4).

#### Case #2: Female, aged 57 years

##### Symptoms and clinical diagnosis: amyotrophic lateral sclerosis

This ALS patient was one of the neurodegeneration parts of the 449 patient group, where the R/C overexpression in FDOJ was supposed to be part of the systemic disease [11, 13].

**Fig. 5:**
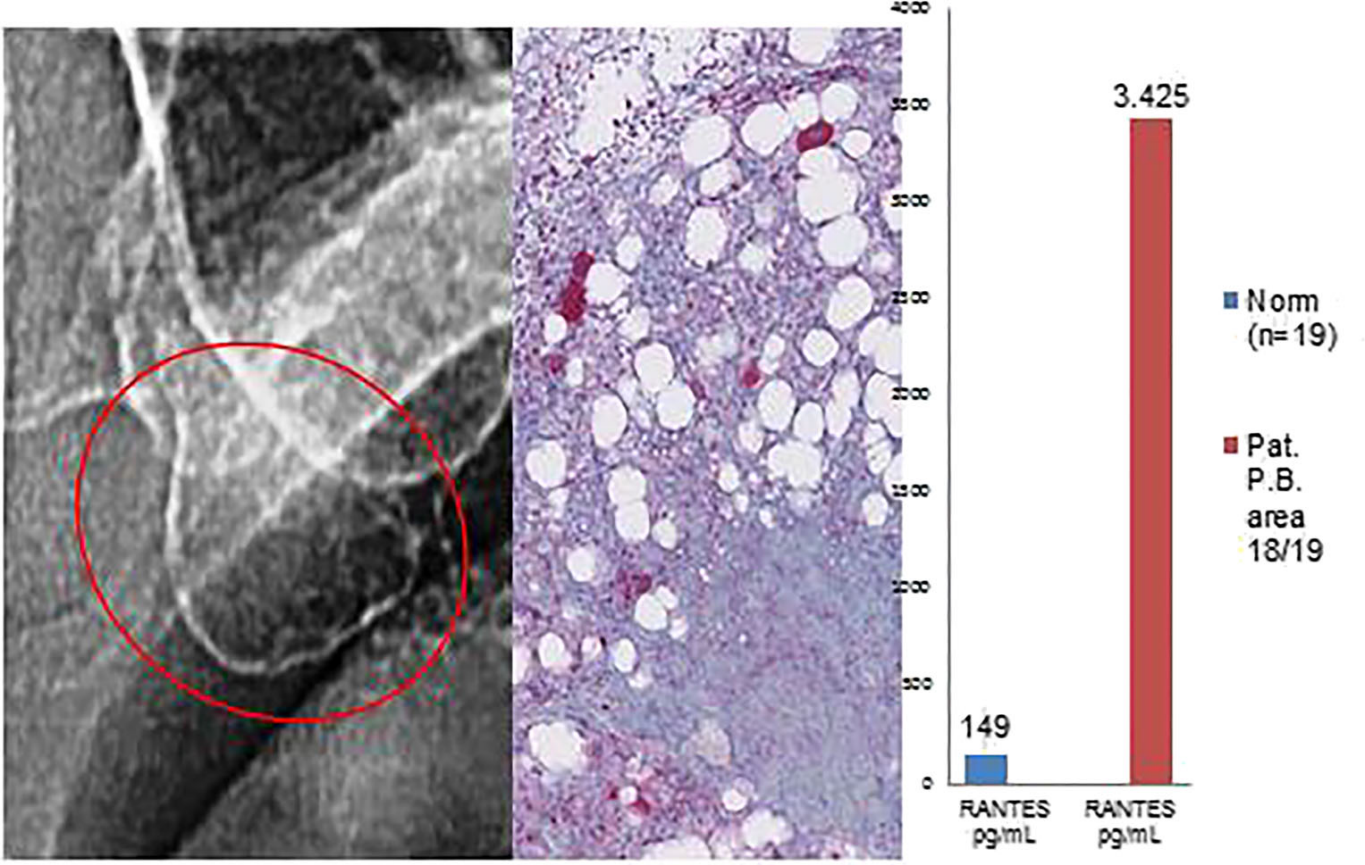
Left panel: Inconspicuous, two-dimensional X-ray diagnostics of area 18/19 with a bone density of − 212 HU. Middle panel: Immunohistochemistry shows a strong, red stain for adipocytogenic R/C overexpression. Right panel: Multiplex R/C expression at a level of 3425 pg/mL (norm = 149 pg/mL)

Histological diagnosis and assessment: Medullary tissue from jawbone area 18/19 with exclusively fatty regions which is characterized by loose fibrosis and necrobiotic adipocyte changes. There is also focal evidence of increased bone remodeling of the bone trabeculae, as well as mucinous adipose tissue changes. These findings are suggestive of FDOJ (Fig. 5).

### Clinical cases (group 2) from the jaw area—comparison of RANTES/CCL5 overexpression and negative histology

#### Case #3: Female, aged 44 years

##### Symptoms and clinical diagnosis: chronic fatigue syndrome

**Fig. 6:**
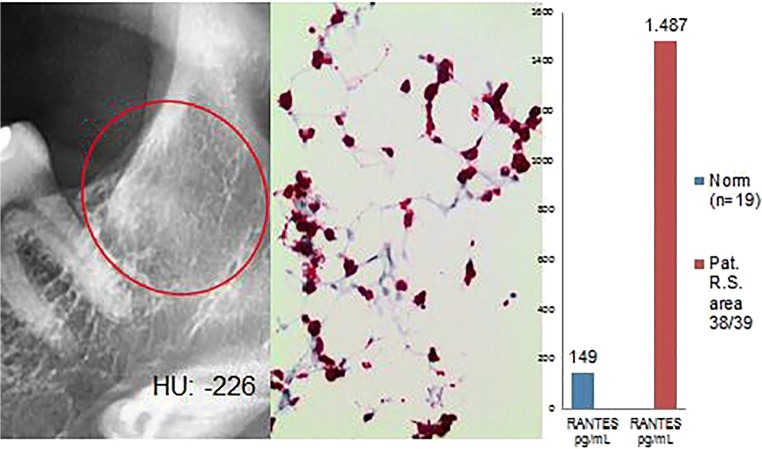
Left panel: Inconspicuous, two-dimensional X-ray diagnostics for area 38/39 with a bone density of − 226 HU. Middle panel: Immunohistology with antibodies against CD15 shows a clear overexpression of CD-15-postive cells; thus, there is a markedly left-shifted marrow indicating the presence of inflammatory marrow. Right panel: Multiplex R/C expression at a level of 1487 pg/mL (norm = 149 pg/mL)

Histological diagnosis and assessment: Medullary tissue from jawbone area 38/39 with regular hematopoietic marrow in three bands; sparse bone trabeculae without obvious pathological changes. In this sample, therefore, there is no indication of FDOJ (Fig. 6).

#### Case #4: Male, aged 28 years

##### Symptoms and clinical diagnosis: chronic fatigue syndrome; atypical facial pain

**Fig. 7:**
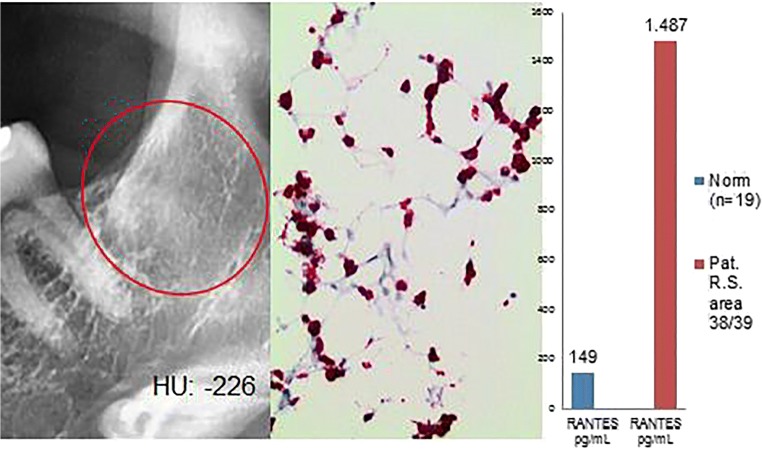
Left panel: Inconspicuous, three-dimensional DVT X-ray of area 38/39 with a bone density of − 117 HU. Middle panel: Immunohistology with antibodies against CD-15 shows a significant overexpression of CD-15-positive cells, thus demonstrating a markedly left-shifted marrow which suggests the presence of inflammatory marrow. Right panel: Multiplex R/C expression at a level of 5587 pg/mL (norm = 149 pg/mL)

Histological diagnosis and evaluation: A sample from jawbone area 38/39 with blood-forming marrow. The structured bone trabecula does not feature any abnormalities. There is no evidence of FDOJ in this sample (Fig. 7).

### Clinical cases (group 3) from the jaw area—reduced RANTES/CCL5 expression and normal histological findings but inflammation-related alterations in the bone marrow

From the 449 FDOJ samples, 14 cases showed reduced R/C expression along with a common histological finding that was not indicative of FDOJ, despite an HU < 150 (i.e., class 5) and clinically/macroscopically clear osteolysis at the surgical site. Once again, the preparations were subjected to immunohistological staining to assess R/C expression. Using immunohistology, we aimed to elucidate whether inflammation-altered bone marrow is, in fact, present in these cases and the hematopoietic marrow is left-shifted (i.e., indicative of inflammation).

#### Case #5: Female, aged 57 years

##### Symptoms and clinical diagnosis: amyotrophic lateral sclerosis

**Fig. 8:**
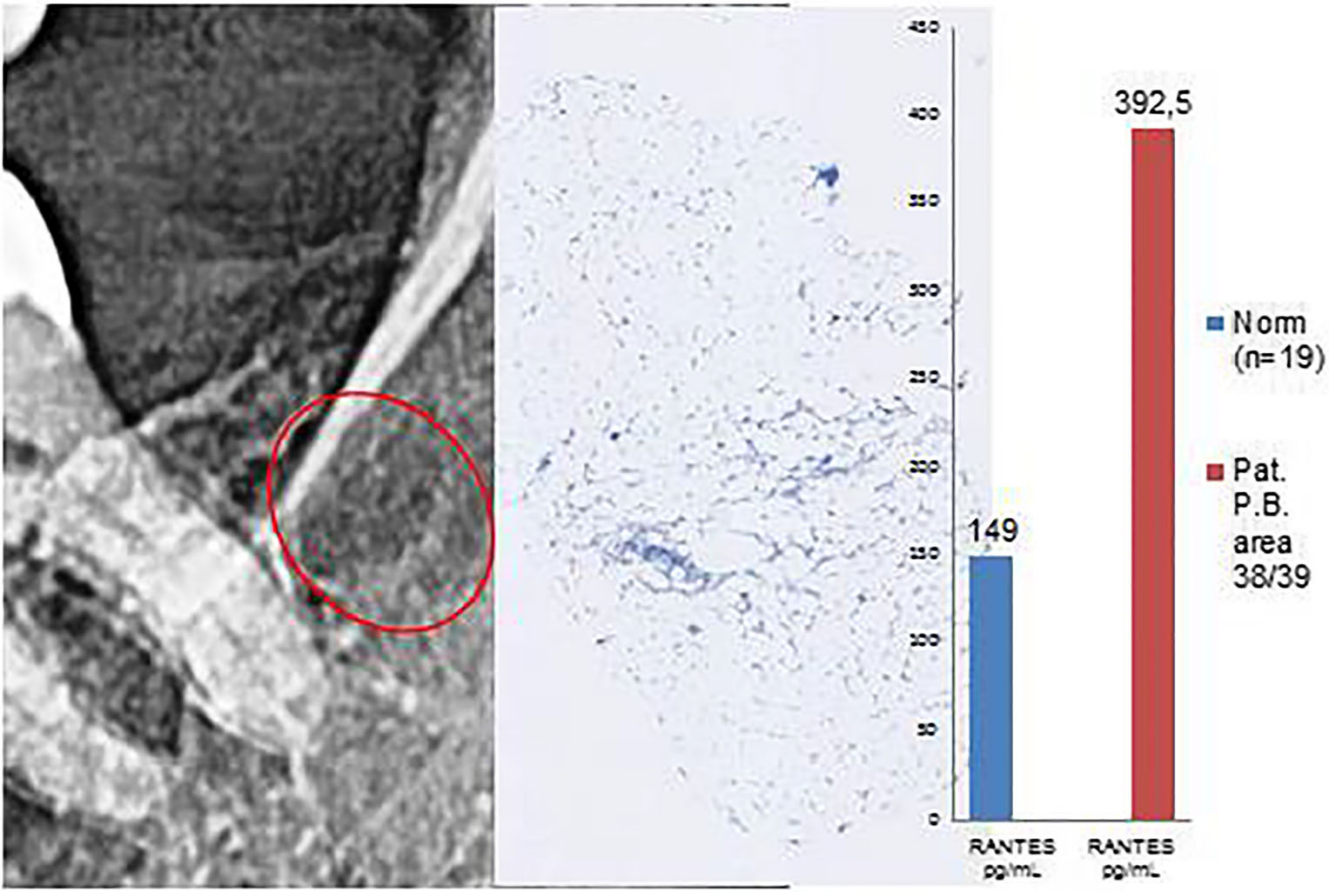
Left panel: Inconspicuous, two-dimensional X-ray of area 38/39 with a bone density of − 64 HU. Middle panel: Immunohistochemistry does not show any red staining for adipocytogenic R/C overexpression. Right panel: Multiplex R/C expression with atypical levels of 392.5 pg/mL (norm = 149 pg/mL)

**Fig. 9:**
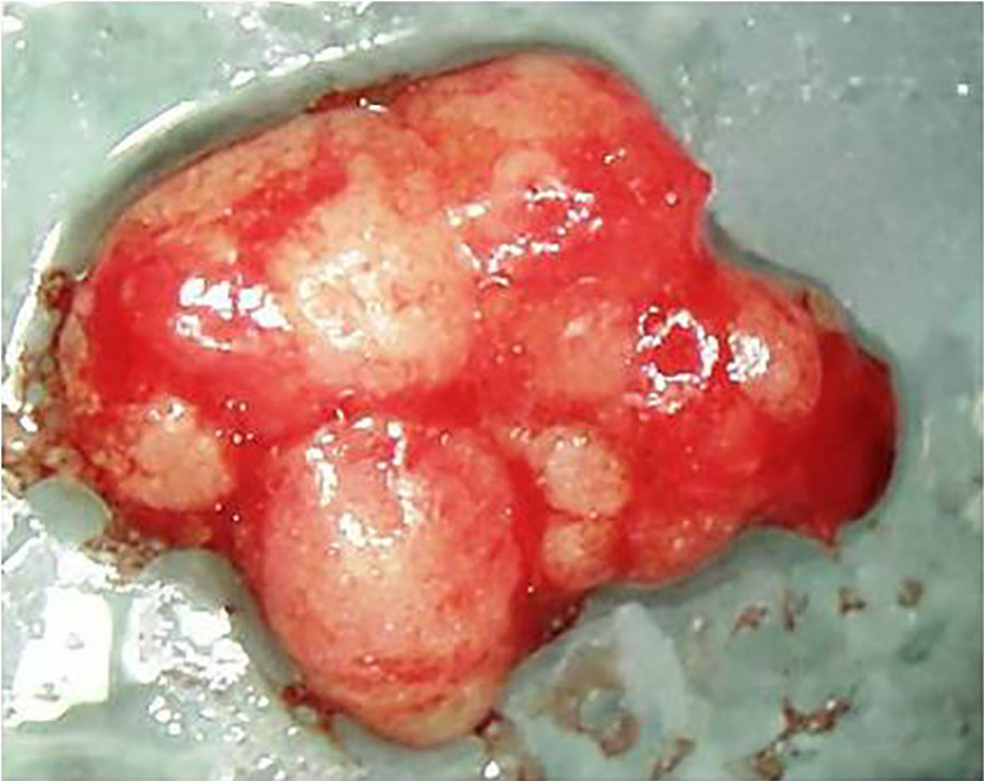
Macroscopic view of the fatty degenerative medullary portion from area 38/39. A typical FDOJ structure with complete resolution of the spongy microarchitecture of the jawbone is evident, in contrast with standard histological findings and the absence of staining in the immunohistochemistry analysis with a correspondingly low R/C expression level

Histological diagnosis and evaluation: Medullary tissue from jawbone area 38/39 with predominantly fatty marrow; this tissue is without pathological changes and features regular adipocytes and intact cell membranes and is thus suggestive of normal findings. There is no evidence of FDOJ (Figs. 8 and 9).

#### Case #6: Female, aged 29 years

##### Symptoms and clinical diagnosis: rheumatic arthritis

**Fig. 10:**
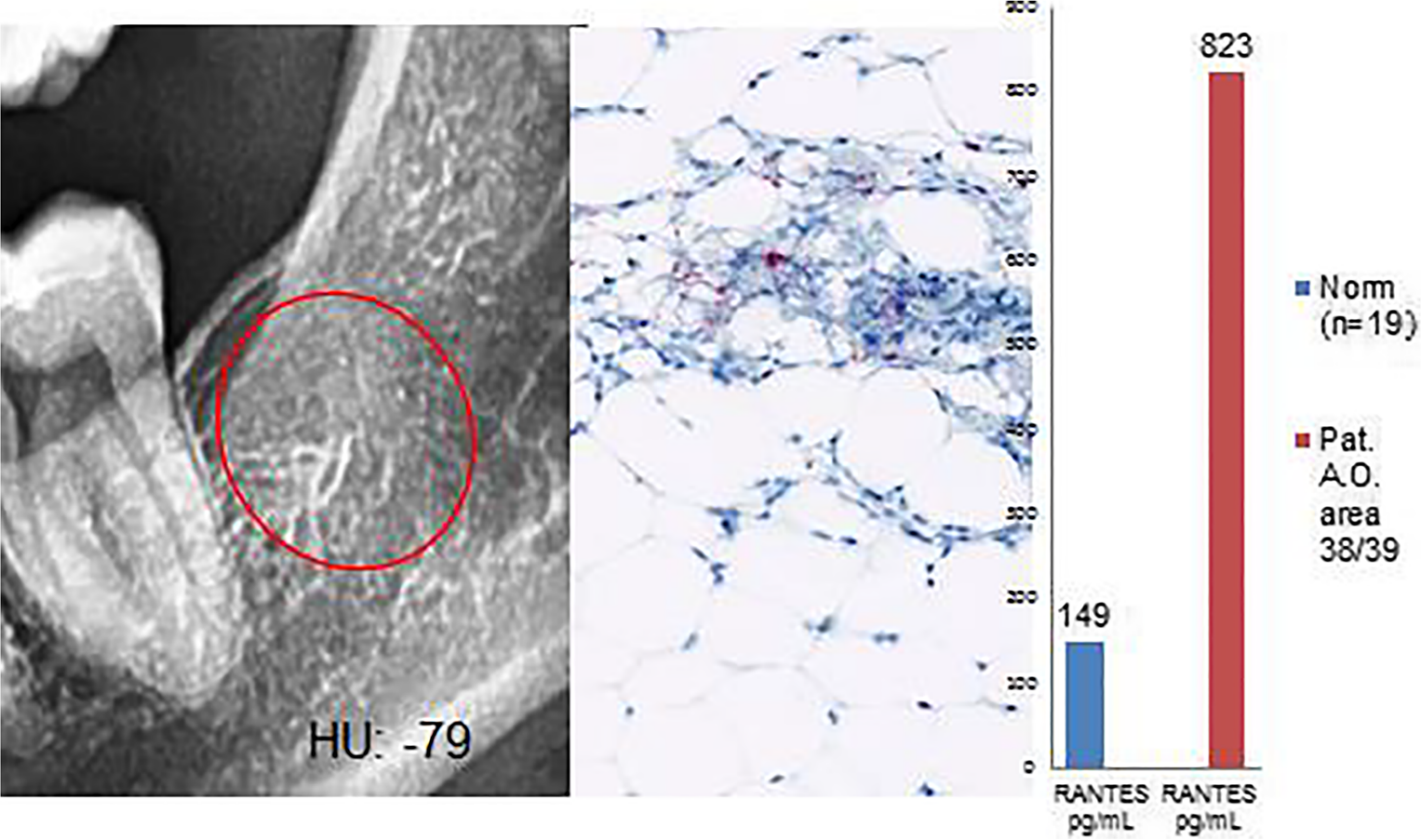
Left panel: Inconspicuous, two-dimensional X-ray of area 38/39 with a bone density of − 79 HU. Middle panel: Immunohistochemistry shows weak, red staining for R/C expression, primarily in the megakaryocytes of the blood marrow. Right panel: Multiplex R/C expression with atypically low levels of 823 pg/mL (norm = 149 pg/mL)

Histological diagnosis and assessment: Medullary tissue from area 38/39 with exclusively hematopoietic marrow. The adipocytes are distributed regularly in the hematopoietic marrow without abnormalities; furthermore, there are no indications of increased bone remodeling. Thus, this tissue indicates histologically normal findings without the presence of any repair mechanisms, despite a bone density of – 79 HU (Fig. 10).

### Summary of results

**Fig. 11:**
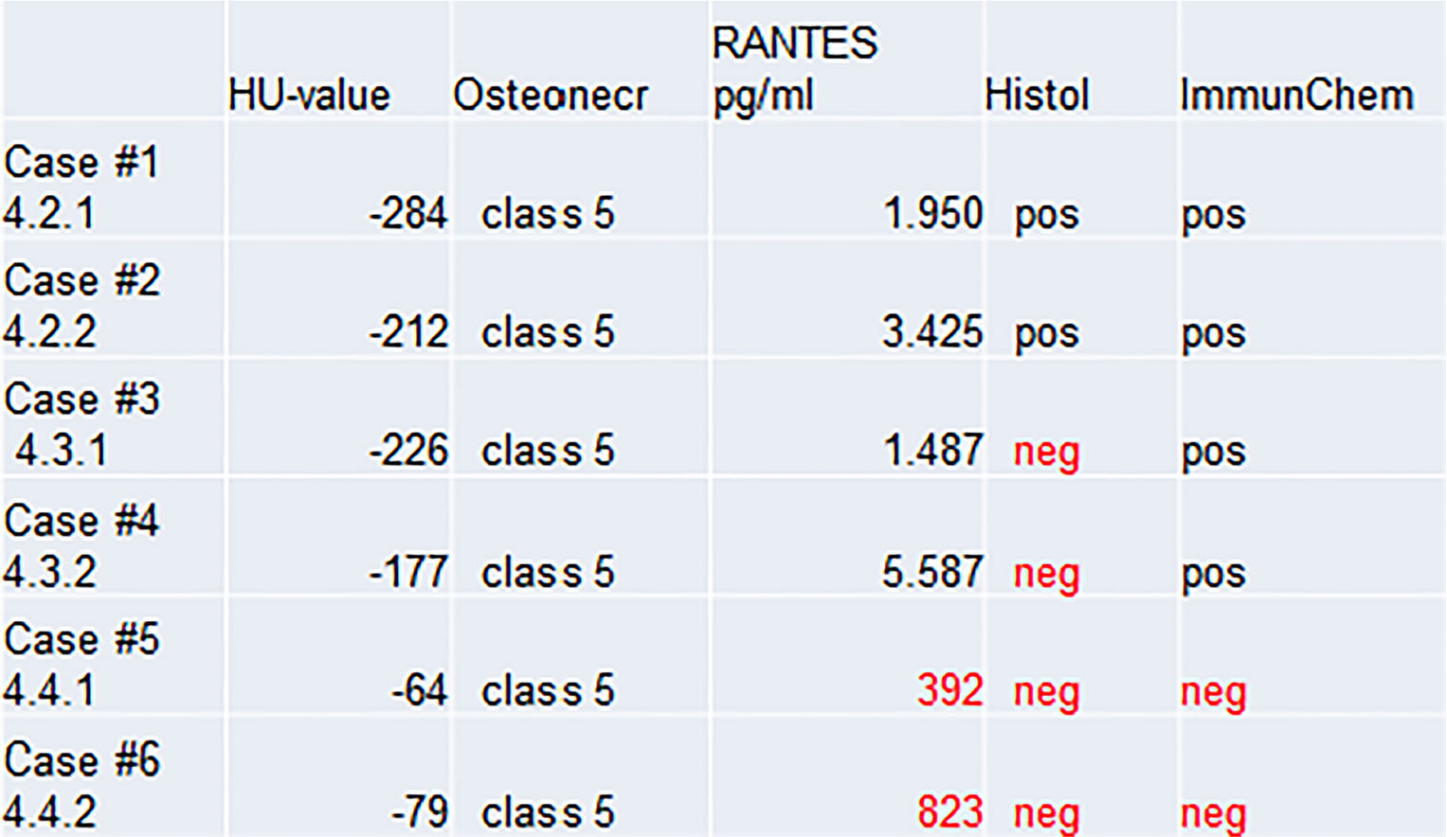
Comparison of HU bone density and osteonecrosis to RANTES/CCL5 expression, histology, and immunohistochemistry findings in all six clinical case studies

Figure 11 provides a summary of the results. All FDOJ areas that were surgically removed were osteolytically softened, as indicated by the decreased bone density measured in HU (< 150; class 5). The FDOJ samples from multiple cases show R/C overexpression (< 850), which is also consistent with the corresponding immunohistochemistry findings. However, R/C expression does not always correlate with histological or immunohistochemical findings.

## Discussion

Following the recommendations of PPPM, individually targeted prevention is a crucial part of this diagnostic and therapeutic concept. The adipose tissue associated with FDOJ is highly ductile and seemingly pliable. The adipocytes which comprise this tissue frequently contain a single, large liquid vacuole consisting of triglycerides (i.e., oil cysts). However, an adipocyte is not merely a passive “fat reservoir”; it also actively produces and secretes proinflammatory cytokines. The aim of this study was to determine the extent to which the cytokine patterns of pathologically altered jawbone medulla derail OC and OB homeostasis, thus impacting the remodeling activity in those areas affected by FDOJ. We also sought to investigate the extent to which the current literature was able to shed light on the local histological, osteogenic, adipogenic, and immunological situations in areas affected by FDOJ. Following this, we aimed to determine the extent to which the lack of agreement in our histological and mediator-based findings could be explained. Concurrently, we wished to draw attention to the possible systemic interactions of bone remodeling with the immune system, an area of research which may result in a new concept known as “maxillo–mandibular osteoimmunology”.

Focal points of hematopoietic marrow, which persist in certain areas of the jaw until adulthood, are usually limited to the condylar process, the angle of the mandible, and the maxillary tubercle [18, 19]. The etiology of focal osteolysis of the bone marrow remains poorly understood and may be caused by any of the following: (a) the persistence of fetal bone marrow; (b) altered trabecular repair in an area of trauma or inflammation; (c) an increased systemic need for blood cells that stimulate the development of hematopoietic bone marrow foci; or (d) an ischemic change in the bone marrow tissue [20, 21]. The radiographic appearance of these defects is not sufficiently characteristic to allow for an accurate diagnosis. Circumscribed “focal” bone defects in the jawbone, (i.e., “focal osteoporotic marrow defects”), are the subject of scientific presentations and discussions [22]. We have consistently found that in areas of the jawbone which remain incompletely healed, fatty degenerative osteolysis of the medullary spongiosa develops; these areas are pathohistologically characterized by fibrosis and adipocyte degeneration [23]. In conjunction with the presence of fat cells in the jaw, we were able to detect the overexpression of the inflammatory chemokine, R/C, in multiplex examinations of edentulous areas of the jaw. Contrary to numerous studies found in the literature which are usually performed on mice, we measured R/C levels in vitro in the jawbone of patients with immune system diseases [6]. R/C is expressed in a wide range of immune and non-immune cells in response to stress signals. It is known that R/C acts as a chemoattractant for differentiated cells such as monocytes, eosinophils, T cells, natural killer cells, and dendritic cells. R/C is released by stromal cells in damaged tissue; it binds to glycosaminoglycans at the endothelium and, via diapedesis, engages the immune system by calling on peripheral blood cells to sites of inflammation. Activated T cells express R/C 3–5 days after stimulation, thus enhancing the immune response.

### Why is RANTES/CCL5 overexpressed by adipocytes? (With reference to the clinical cases presented in “Clinical cases (group 1) from the jaw area—comparison of X-ray diagnostics, R/C expression, light microscopy, and immunohistochemistry“)

Cases 1 and 2 show the characteristic findings of FDOJ which include inconspicuous X-ray imaging in a two-dimensional OPG, decreased bone density (as indicated by HU on three-dimensional DVT, as was evident in case 1), clinical osteolysis, and postoperative FDOJ histology with immunohistochemically positive staining for R/C overexpression. This raises the question of whether there is a link between R/C overexpression and degenerated fatty acids. Fat marrow itself is not pathological; almost all adult bones have high fat levels. Fatty changes only become pathological when the fatty marrow disintegrates. The bone trabeculae thin out and the balance between osteogenesis and adipogenesis increasingly shifts toward the production of adipocytes. The supporting bone trabeculae disappear and fatty areas are impacted by secondary damage which eventually leads to the development of oil cysts as previously described by us and other authors [6, 20]. The microarchitecture of bone is thus an important parameter that can be used to describe the chronically disturbed process of remodeling and OC resorption [23]. The local accumulation of resorption lacunae through enhanced OC activity may result in an irreparable structural loss by decoupling OC bone resorption from OB bone augmentation. If this process continues for an extended period of time, it may result in the conspicuous macroscopic morphology of FDOJ, as described earlier. These changes in the microarchitecture of FDOJ are typically found in post-traumatic changes in the jaw [1]. When compared with healthy reference tissues, the cytokine genes of R/C are predominantly overexpressed in areas of FDOJ. Macrophages and fibroblasts—as target cells and producers of R/C—appear to be strongly involved in the degenerative process associated with FDOJ.

### Why does “blood formation/hematopoiesis” results in fatty degeneration of the medulla in the jawbone? (With reference to the clinical cases presented in “Clinical cases (group 2) from the jaw area—comparison of RANTES/CCL5 overexpression and negative histology” and “Clinical cases (group 3) from the jaw area—reduced RANTES/CCL5 expression and normal histological findings but inflammation-related alterations in the bone marrow”)

Some of the 449 FDOJ cases we examined showed superficial inconsistencies between light microscopy findings and all other parameters. At first glance, the light microscopy findings and conspicuously high R/C expression levels seem to contradict one another. This apparent conflict, however, is resolved by examining in greater detail the supposedly inconspicuous bone marrow biopsies from an immunohistological perspective. In fact, pathological immunohistological changes were found in the cases with “negative” light microscopy findings in the group presented in “Clinical cases (group 3) from the jaw area—reduced RANTES/CCL5 expression and normal histological findings but inflammation-related alterations in the bone marrow.” This group showed alterations in the form of inflammatory, reactive bone marrow. This finding is consistent with the persistent irritation that is associated with increased hematopoiesis in the early stages of FDOJ. The assumption that this finding represents the initial stages of inflammation seems justified as blood is typically involved in the irregular healing process which occurs in FDOJ, leading to the typical features at its chronic end stage. Despite focal osteolysis and predominant R/C overexpression, hematopoietic marrow is found in FDOJ. In the resulting inflammatory reaction, excessive R/C mediators are released; these are no longer able to be broken down, thus prolonging the intramedullary inflammatory response. The dysregulation that occurs in the initial stages of inflammation, followed by subsequent degeneration, fits well with the fact that many degenerative diseases begin as inflammatory processes that ultimately become chronic and lead to degenerative atrophy, as in Sudeck’s dystrophy. It is well known that adult jawbone no longer contains blood-forming marrow. In adults, very small blood foci are present in the marrow, (e.g., in the sternum). One must ask, however, why these blood focus findings are lacking in cases 1 and 2. Similarly, immunostaining showed high R/C expression in cases 3 and 4. The question arises as to whether this represents subtle, mostly unnoticed hematopoietic marrow in the context of chronic irritation. Additionally, one may ask whether it is possible that this high R/C level represents a chronic inflammatory process in the fat marrow of the jawbone, suggestive of a level of irritation that can be stimulated via blood formation. When this chronic irritation is prolonged for years, for instance, the question then also arises as to whether blood formation will return to normal or be characteristic of typical FDOJ. The focus of our study is thus on the effects and possible key functions of overexpressed R/C in areas affected by FDOJ in order to further the discussion concerning the interactions of FDOJ with hematopoiesis.

#### The hematopoietic stem cell niche and RANTES/CCL5 in the bone marrow

Blood cells, (e.g., erythrocytes), develop from HSCs through cell division and increasing differentiation. From multipotent HSCs, the lineages of myeloid and lymphatic hematopoiesis are developed. Under the influence of cytokines, the morphologically distinct precursors (“blasts”) of the various blood cells mature from HSCs. HSCs are, as it were, the “primordial cells” of blood formation [24]. The fate and development of HSCs are largely determined by the cellular environment in the bone marrow which consists of a mixture of stromal and endothelial cells, OBs, and adipocytes [25]. Our primary interest is the factors that determine the fate of HSCs, such as the self-renewal or differentiation of the bone marrow. HSCs must continuously make correct decisions in order to meet current regeneration requirements. These cells are significantly influenced by their environment (niche) within the bone marrow; soluble factors, cell–cell interactions, and contact with the extracellular matrix play a crucial role, as well as intrinsic signals. In homeostasis, HSCs maintain the current cell count in the peripheral blood and tissues. Constantly aging and dying cells are replaced by new ones. The different instructive or selective effects of exogenous signals and intrinsic factors impact upon the decisions of HSCs [26, 27].

#### RANTES/CCL5 affects hematopoietic stem cell subtypes and causes myeloid imbalance

What role does R/C play in influencing the behavior of HSCs? These cells undergo dramatic changes with age. A hallmark of hematopoietic aging is an increase in the number of HSCs; this is also associated with a functional deficit in their reconstitution potential and results in a shift toward the production of myeloid cells. In older mice, the potency of HSCs decreases and they also experience a decrease in immune function and an increased incidence of myeloid malignancies [28, 29]. At the same time, high concentrations of R/C are found in the aging stem cell milieu. Ex vivo exposure of HSCs to R/C results in a decrease in T cell progeny; this is associated with an increase in myeloid progenitors [30]. These data indicate the role of FDOJ in the development of a myeloid imbalance phenotype; immunodeficiency may also play an important role. Additionally, an inflammatory environment may affect the survival and differentiation of HSCs. In the age-related mouse biology of HSCs, R/C was identified as a key factor [30]. R/C was also identified as an important proinflammatory cytokine in this HSC shift; R/C accumulates in the aged bone marrow and is expressed by both stromal and differentiated blood cells [31]. R/C is released from stromal cells in damaged tissue, binds to glycosaminoglycans at the endothelium, and draws peripheral blood cells from the immune system to sites of inflammation. Activated T cells express R/C within 3–5 days of stimulation thus enhancing the immune response [31]. Exposing HSCs to R/C seems to have a direct impact on the fate decisions of both stem and progenitor cells; therefore, the overexpression of R/C may mimic the aging environment and promote lineage strain [30].

R/C induces the expression of promyeloid transcription factors which are important for the maintenance and expansion of multipotent progenitor cells and HSCs [32]. R/C also reduces the expression of lymphoid-specific genes in progenitor cells that regulate early lymphocyte and T cell development. When exposed to R/C overexpression, stem and progenitor cells divert the process to myeloid differentiation at the expense of lymphoid differentiation [33]. A better understanding of the significant role played by R/C in the aging process and the associated factors may ultimately contribute to alleviating some of the hematopoietic effects of the aging process.

#### Osteolysis in the burned-out end stage: why is there a reduction in RANTES/CCL5 overexpression?

Cases 5 and 6 reinforce the lack of agreement between the findings in cases 4–6 (RANTES/CCL5 affects hematopoietic stem cell subtypes and causes myeloid imbalance); in addition to the microscopic findings where normal tissue was evident, there was a significant reduction in R/C expression compared with other areas affected by FDOJ. One explanation is that these two cases represent the complete exhaustion of all adipocytogenic and osteogenic activities; the cells involved, such as macrophages and fibroblasts, may be in a state of functional fatigue and are thus effectively “burned out”. The osteolytic processes involved have previously been described as morphologically distinct FDOJ and represent the last stage of a regenerative stasis within the area affected. The lack of histological and mediator-based evidence found postoperatively does not necessarily imply that there is a false or incorrect indication for surgical intervention; rather, a positive FDOJ process, established across all four parameters at area 18/19 in case 3 (section group 2), is also evident in case 5 (section group 3) at area 38/39 which presented with a negative histology and immunochemistry. This clarifies the internal dynamics and structures involved in the different phases of osteolysis/osteonecrosis found in FDOJ.

### Four phases of FDOJ development with different R/C expressions

An area of FDOJ with a bone density of less than 150 HU, or a negative bone density measurement with TAU displaying orange or red coloration, develops in four phases. (a) In Phase 1, the onset of osteolysis occurs but low systemic activity corresponds with low R/C expression, poor histology, negative immunohistochemistry but positive radiographs in HU. This phase is exemplified by case 5. (b) In Phase 2, there is marked osteolysis and the onset of systemic activity which shows high R/C expression but no conspicuous histology, i.e., normal bone marrow which may present as left-shift reactive inflammatory marrow. This pre-existing R/C expression corresponds to a positive immunohistochemistry; there is also a positive radiographic finding in HU. This phase is exemplified by cases 3 and 4. (c) In Phase 3, there is a significant systemic effect which shows excessive R/C expression, markedly positive histology, strong positive immunohistochemical staining, and positive radiographic findings in HU. This phase corresponds to cases 1 and 2. (d) Phase 4 shows the final stage of active FDOJ with burned-out R/C expression, corresponding to a negative immunohistochemistry result and decreasing systemic action. Extensive osteolysis remains present with high negative HU values. This phase corresponds to case 6.

### Summary

The case studies presented here show that there is no “typical state of FDOJ” that is clearly defined by light microscopy, immunohistochemistry, or cytokine patterns. Rather, this bone-resorbing phenomenon which occurs in the jawbone is a fluid biological process centered around the large volume, fatty degenerative dissolution of the medullary regions in conjunction with R/C overexpression. The only parameters that are consistent in this study across all four stages of FDOJ are the measurement of bone density at < 300 HU and the fatty degenerative, softened morphology. All functional parameters—cellular structures of histology, R/C expression and, consequently, the immunohisochemistry intensity—indicate ongoing changing states.

## Conclusions—maxillo–mandibular osteoimmunology”—interactions between bone and the immune system in the focus of an individualized and personalized medical approach

Osteoimmunology is an emerging field of research that studies the interactions between the immune and skeletal systems. We have shown that R/C expression primarily regulates immune and bone cell function and influences skeletal health. By presenting data on R/C expression in the medullary jawbone, we hope to contribute to the development of a new understanding of bone marrow as well as the hematopoietic and immune systems. R/C overexpression in FDOJ may be identified as a mechanism that proceeds in different and highly individual immunological phases, and directly influences the decisions of stem cells which result in the chronic and negative manipulation of hematopoiesis. This provides pathogenetically relevant explanations for aberrations and faulty programming in stem cell expansion. We were able to identify the chemokine, R/C, as a key factor within the field of “maxillo–mandibular osteoimmunology” and have described it as such in several papers [1, 6, 7, 13, 34]. For the benefit of systemically ill patients, it is necessary that medicine and dentistry devote further attention to the concept of “maxillo–mandibular osteoimmunology” and the associated personalized signal patterns. The here presented data and pathohistological figures are especially relevant to the objectives of PPPM [35]. A particularly subtle and chronic inflammatory process in FDOJ bone marrow defects may occur in certain individuals and contribute to derailed tissue patterns. In FDOJ, areas emerge typical individual mediator-related signaling patterns of inflammatory cytokines, which describe a step toward further multidisciplinary considerations for personalized dentistry and prevention [36]. Our here achieved results should be used for follow-up research activities and highlight the need for a multi-center prospective study with clearly defined cohorts in order to reliably clarify the causative R/C pathways involved.

## Abbreviations

CBCT: Cone-beam computed tomogram
CCR5: Chemokine receptor 5
CI: Chronic inflammation
CT: Computed tomography
DVT: Digital volume tomogram
HSC: Hematopoietic stem cell
HU: Hounsfield units
MSC: Mesenchymal stem cell
OB: Osteoblast
OC: Osteoclast
OPG: Orthopantomogram
PPPM: Predictive preventive personalized medicine
R/C: RANTES/CCL5
TAU: Transalveolar ultrasonography
18/19: Upper right wisdom tooth area (European Code on Dental Procedures and Nomenclature; US CDT code = #1)
28/29: Upper left wisdom tooth area (US CDT code = #16)
38/39: Lower left wisdom tooth area (US CDT code = #24)
